# Priorities and needs for research on urban interventions targeting vector-borne diseases: rapid review of scoping and systematic reviews

**DOI:** 10.1186/s40249-016-0198-6

**Published:** 2016-12-01

**Authors:** Clara Bermudez-Tamayo, Olive Mukamana, Mabel Carabali, Lyda Osorio, Florence Fournet, Kounbobr Roch Dabiré, Celina Turchi Marteli, Adolfo Contreras, Valéry Ridde

**Affiliations:** 1 Andalusian School of Public Health, Campus de la Cartuja s/n, Granada, 18010 Spain; 2 CIBERESP–Ciber de Epidemiología y Salud Pública, Av. Monforte de Lemos, 3-5. Pabellón 11. Planta 0, Madrid, 28029 Spain; 3 CHU Sainte-Justine, 3175, Chemin de la Côte-Sainte-Catherine, H3T 1C4 Montreal, QC Canada; 4 McGill University, 845 Sherbrooke Street West, H3A 0G4 Montreal, QC Canada; 5 Universidad del Valle (UDV), Cl. 13 #100-00, Cali, Valle del Cauca Colombia; 6 IRD, Research Unit: MIVEGEC (Maladies infectieuses et vecteurs : écologie, génétique, évolution et contrôle), 911, avenue Agropolis, Montpellier, France; 7 IRSS, Bobo-Dioulasso, 399, Avenue de la Liberté, Direction Régionale de l’Ouest, Bobo-Dioulasso, Burkina Faso; 8 Centro de Pesquisa Aggeu Magalhâes – Fiocruz/Pernambuco, Av. Professor Moraes Rego, s/n, Campus da UFPE, Cidade Universitária, CEP: 50.740-465 Recife, PE Brazil; 9 University of Montreal Public Health Research Institute (IRSPUM), 7101, Avenue du Parc., Montreal, H3N1X9 Quebec Canada; 10 Department of Social and Preventive Medicine, School of Public Health, University of Montreal, 7101, Avenue du Parc., Montreal, H3N1X9 Quebec Canada

**Keywords:** Vector-borne diseases, Urban health, Intervention

## Abstract

This paper highlights the critical importance of evidence on vector-borne diseases (VBD) prevention and control interventions in urban settings when assessing current and future needs, with a view to setting policy priorities that promote inclusive and equitable urban health services. Research should produce knowledge about policies and interventions that are intended to control and prevent VBDs at the population level and to reduce inequities. Such interventions include policy, program, and resource distribution approaches that address the social determinants of health and exert influence at organizational and system levels.

**Electronic supplementary material:**

The online version of this article (doi:10.1186/s40249-016-0198-6) contains supplementary material, which is available to authorized users.

## Multilingual abstracts

Please see Additional file 1 for translations of the abstract into the five official working languages of the United Nations.

## Background

Currently, more than half of the world’s population lives in urban areas, and this proportion will grow to almost 70 % by 2050, according to a recent UN report; the largest increases will occur in Asia and Africa, accounting for 90 % of this growth [1]. Urban life is generally characterized by better quality of life for the population, in terms of access to health services, social and educational services [2]. The other side of this reality, however, is that unplanned urbanization and migration lead to concentrations of the poorest and most vulnerable, making the urban environment itself a social determinant of health.

Urbanization poses a major public health challenge for the 21st century, and several health outcomes correlate with urbanization processes, particularly in low- and middle-income countries (LMICs) [3]. Urban regions can become breeding grounds for emerging and re-emerging communicable diseases. Possible factors associated with this are the ecology of urban environments, poverty, population mobility, and microbial adaptation to changes [4, 5]. Vector-borne diseases (VBD) have been found to be increasing in cities and urban areas, making them “gateways for the worldwide spread of infections” [6]. As noted by Imperato, “The Zika virus epidemic is the latest in a recent series of globalized emerging infections. During the past decade, epidemics of Dengue, West Nile, Chikungunya, and Ebola have spread out of what was once assumed to be their restricted geographic spaces” [7]. With globalization, infections are more than likely to appear and reappear, which will require public health interventions to curb their spread and minimize their effects on global population health [7].

LMICs have some of the highest VBD risks through increased exposure to disease agents and greater vulnerability to infection. The global and local programs created to mobilize resources for these diseases need to employ multi-sectorial and multi-level integrated approaches, positioning VBDs within the social determinants of health framework. Moreover, interventions to prevent and control VBDs must address the contributory factors and the three interacting aspects of daily urban life: the physical environment, social conditions, and climate change.

Context-relevant and proven interventions for VBD prevention and control are critical for assessing current and future needs, with a view to setting public health policy priorities. In this respect, a recent UN report noted that, “The outcome of the Rio + 20 United Nations Conference on Sustainable Development, ‘The Future We Want,’ recognized both the plight of the urban poor and the need for sustainable cities as matters of great urgency for the United Nations development agenda. Building on that momentum, the third United Nations Conference on Human Settlements (Habitat III) is planned for 2016, to bring together world leaders to review the global urban agenda and forge a new model of urban development that integrates all facets of sustainable development to promote equity, welfare and shared prosperity in an urbanizing world” (p. 3) [1]. Accurate, consistent, and evidence-based interventions for VBD prevention and control in urban settings are needed to implement public policy and to promote inclusive and equitable urban health services. In this article we highlight this importance by briefly reporting the results of a narrative rapid review of evidence on health interventions for VBD prevention and control.

## Health interventions for the prevention and control of VBD

Based on a rapid review of scoping and systematic reviews performed in this field (methodological details in Additional file 2), we examined the evidence, grouped into seven themes (Table 1), pertaining to urban health interventions for the prevention and control of VBD in LMICs listed by the World Bank [8]. To capture the breadth of the evidence and to identify potential gaps in the topics, we used the already established themes from the WHO/TDR call (September 2015) as a starting point for the rapid review [9].

**Table 1:** Themes relating to urban health interventions for the prevention and control of VBD

1. VBD transmission dynamics in urban settings
2. Evolution of urbanization trends and VBD
3. Governance issues influencing urban VBD control
4. Issues in and solutions for infectious disease prevention and control related to housing, water and sanitation
5. Implementation research findings on infectious disease prevention and control in urban settings
6. VBD surveillance and community-based risk communication in urban areas
7. Population dynamics and their interaction with social determinants of health

Systematic and scoping reviews were the focus of the searches because of their methodological strength, which made it possible to explore the extent and nature of the evidence and to analytically interpret and assess the quality of the reviewed evidence.

Overall, the rapid approach yielded several literature reviews lacking in detail regarding the methods used to conduct them, and few systematic or scoping reviews were found for three of the reviewed themes. Here we present some of the main findings and research gaps observed in our review.

### VBD transmission dynamics in urban settings

VBDs have long been presented as diseases of rural areas. However, the ecology of vector systems is complex, being subject to profound and rapid changes (alterations in the natural environment, development of agro-pastoral practices, urbanization, development of transport, climate change). As such, it is important to develop a good understanding of vector systems in their context, which involves adopting an ecological approach to disease and requires the cooperation of many specialists in diverse fields such as microbiology, virology, parasitology, entomology, climatology, ecology, urban planning, social science, political science, and public health [10].

The ecology and epidemiology of VBDs are particularly complex and often involve multiple disease cycles through alternate vectors and hosts. To investigate infectious disease outbreaks, it is important to determine the route of transmission, and in the case of VBDs, to focus on the vector, and in particular its presence, abundance, and ecology. It is also essential to study the influence of environmental conditions, as well as factors related to human behaviour, genetics, and pathogens [11].

Research in this field was first oriented toward analyzing rural–urban differences, then toward studying differences between urban and suburban settings, and more recently to evaluating the existence of intra-urban spatial disparities. These disparities relate to the effects of places, population behaviour, and urban architecture [12].

The epidemiological pattern of urban VBDs can change, compared to what was known in rural areas. This evolution is linked to a different human–vector contact (less narrow than in rural areas) and access to different methods of prevention and control (better access to mosquito nets and to healthcare in cities).

Regarding VBDs themselves, malaria has clearly been a major research subject, but arbovirus infections are becoming increasingly important in terms of their emergence in the world [13].

#### Main findings and observed research gap

We identified one systematic review that studied the effect of urbanization on malaria transmission and the burden of this disease in Africa [14]. That study indicated that more evidence is needed to provide scientists and policymakers with new opportunities to quantify and perhaps predict the interaction between the characteristics of urban development and the natural history of diseases in LMICs over time. The Rapid Urban Malaria Appraisal (RUMA) project, undertaken to systematically study key malariological features [15–19], made it possible to describe transmission patterns in urban areas of sub-Saharan African countries: Abidjan (Côte d'Ivoire), Ouagadougou (Burkina Faso), Cotonou (Benin), and Dar es Salaam (United Republic of Tanzania).

#### Evolution of urbanization trends and VBDs

According to the previously mentioned United Nations report, “Today’s cities are growing twice as fast in terms of land area as they are in terms of population. Consequently, projections indicate that future trends in urbanization could produce a near tripling in the global urban land area between 2000 and 2030, as hundreds of thousands of additional square kilometers are developed to urban levels of density. Such urban expansion threatens to destroy habitats in key biodiversity hotspots” (p. 3) [1].

##### Main findings and observed research gap

The perspective on cities often focuses primarily on slums, where 863 million people were living in 2012, nearly a third of them in urban developing countries. Should the undeveloped districts of Ouagadougou, for example, be considered at risk for disease? Does the threat of VBD exist in these suburban areas? With respect to malaria, the conquest of structured neighbourhoods is real, since anopheles vectors are adapting not only to the urban pollution of their habitats but probably to air pollution as well, and perhaps even to light (mosquitoes are normally active at night, in darkness, but cities are increasingly lit at night, even in developing countries). As for dengue transmission, it is closely linked to urbanization because of the human impact of its vector. Indeed, the construct of dengue as a disease of poverty is not entirely accurate, as evidenced by outbreaks in cities that could be described as rich, in Europe, South America, and Asia [6].

A scoping review was found that summarized the impact of urbanization on the epidemiology of infectious diseases, including some VBDs in tropical countries and the emergence of infectious diseases over time [20]. Given that urbanization increasingly affects the epidemiological characteristics of infectious diseases, more research is needed to study the VBD-specific effects of urbanization.

#### Implementation research findings on infectious disease prevention and control in urban settings

Some studies in this area have shown that good connections between the implementers and the targets of interventions (people and vectors) are determinants of the success of interventions. Identified barriers encountered by community intervention programs to prevent and control dengue include stigmatization of the poor, population non-compliance, and low investment in children as a vehicle for public health messages [21, 22].

We also found a scoping review that used a social determinants of health framework to examine key issues related to the prevention and control of VBDs such as malaria and dengue fever in urban informal settlements [23]. In assessing the impact of disease control and prevention in informal settlements, it is important also to study the interventions’ effects in terms of reducing health inequities and addressing underlying social determinants of health.

The burden of VBD is unevenly distributed, with an overwhelming impact in LMICs, principally in subtropical and tropical areas. “The heterogeneity in exposure to infection can be exploited to optimize vector control such efforts cannot succeed unless they are tailored to the local epidemiological and ecological conditions that influence disease transmission” [9]. Another recent review showed that it would be beneficial for researchers to examine and analyze more closely the characteristics of the context (cultural, political, and economic) within which research is conducted and transfer activities implemented [24]. Their results highlighted the need for “systematic evaluation of the conditions for research results use in the settings where transfer activities occur, to identify strategies that specifically target barriers inherent to the context” [24].

#### Other themes on urban health interventions

##### Main findings and observed research gap

No relevant systematic or scoping reviews were found for the following themes: governance issues influencing urban VBD control; VBD surveillance and community-based risk communication in urban areas; issues in and solutions for infectious disease prevention and control related to housing, water, and sanitation; implementation research findings on infectious disease prevention and control in urban settings; and population dynamics and their interaction with social determinants of health.

Relatively little attention has been paid to research on population health interventions to control and prevent VBDs specifically in urban settings. The corpus of peer-reviewed literature documenting the implementation of VBD prevention and control measures, including the burden of social determinants of health, is limited. The majority of the reviews identified did not focus particularly on urban settings and were concentrated mainly on malaria and dengue.

## Addressing research gaps and priorities

Population health intervention research should produce knowledge about policies and programs to control and prevent VBDs at the population level and to reduce inequities. Operating within and beyond the health sector, these interventions should include policy, program, and resource distribution approaches that address the social determinants of health and exert influence at organizational and system levels. The challenge for VBD prevention and control is therefore not only in identifying which interventions work, but also in considering i) implementation and governance issues, ii) determinants of health, iii) political, social and economic conditions, and iv) the urban context.

To achieve this requisite holistic visualization of the subject, research conducted by interdisciplinary teams in close collaboration with policymakers is needed to support evidence-based decision-making for VBD prevention and control in urban areas. Sustained linkages between researchers and knowledge users are important for knowledge production, translation, and application. Researchers in the field of VBDs and public health can provide scientific input and practical evidence on interventions to control and prevent VBDs, while policymakers and knowledge users can make informed decisions at all levels (Table 2). Hence, intersectoral and interdisciplinary collaboration must be embedded into research activities to incorporate knowledge production and knowledge translation strategies that are essential to successfully prevent and control VBDs in urban settings.

**Table 2:** Selected vector-borne diseases: characteristics, existing control, prevention tools and challenges

Disease	Agent	Vector	Burden	Existing prevention strategies	Challenges for VBD control
Malaria	Parasite (*Plasmodiu*, five species)	Anopheles (more than 60 species)	Transmission in 97 countries. About 3.4 billion people at risk.	Outdoor and indoor residual spraying Bed nets (traditional and long lasting insecticidal nets) Insecticides, repellents. Environmental management: Reducing breeding sites by managing water storage, draining water recipients, cleaning backyards, and waste management Biological control: Introduction of parasites or predators to control de vectors. Genetic control: Use of Wolbachia Chemoprophylaxis: Prophylaxis and preventive therapies, mass treatment, vaccines	Lack of expertise in vector control Limited surveillance Limited sanitation and limited access to safe drinking water Resistance to insecticides Environmental change Limited research on fidelity of implemented measures Lack of intersectoral work
Dengue	Virus (*Flavivirus* = Dengue virus, 4 serotypes)	Aedes Aegypti (same vector for yellow fever, Chicungunya and Zika virus)	More than 100 countries at risk. 2.5 billion people at risk.		
Leishmaniasis Cutaneus (CL); Mucocutaneous (MCL) and Visceral (VL).	Parasites-Protozoa (*Leishmania sp*, more than 20 species)	Sand flies (*Lutzomya*)	1.3 million new cases every year. More than 65 % of CL occurs in six countries. MCL occurs mainly in three countries of the Americas.		
American Trypanosomiasis, Chagas	Parasite (*Trypansosoma cruzi*)	Triatomine bugs	10 million infected people worldwide.		
Human African Trypanosomiasis (sleeping sickness)	Parasite (*Trypanosoma brucei gambiense*)	Flies (tsetse fly)	Occurs in 36 sub-Saharan Africa countries. Yearly cases are under 20 000 and 65 are estimated to be at risk.		
Lyme disease	Bacteria (*Borrelia*)	Ticks (*Ixodes Ticks*)	7.9 cases per 100 000 people in the US. Occurs in Asia, Europe and North America.		

Source: World Health Organization. Vector Borne Diseases. http://www.who.int/campaigns/world-health-day/2014/global-brief/en/

### Limitations

As rapid review products, our evidence summaries inherently are more constrained than systematic reviews, given that they are produced within a short timeframe using limited resources. The methods are somewhat less rigorous than those of a traditional systematic review, and evidence summaries may therefore be subject to more bias and/or error [25]. While research comparing rapid with systematic reviews is limited, some have found that, despite “axiomatic differences” between the rapid and full reviews evaluated, “the essential conclusions of the rapid and full reviews did not differ extensively”, suggesting that this evidence summary may offer a useful and valid approach [26]. In this paper we address VBDs as a broad and general topic without the level of specification that will be required to provide disease-specific or country/region-specific recommendations. Nonetheless, we consider it to be a helpful starting tool whose groupings will undergo better categorization in subsequent studies.

## Conclusion

To prevent and control vector-borne diseases, sustainable urban development must incorporate interventions that consider the social, economic, demographic, and ecological diversity of urban scenarios and the changing biology of vectors. Information needed to lead (or recommend) such interventions could be obtained through the reviews that the consortium intends to produce.

## Additional files

Additional file 1: Multilingual abstracts in the five official working languages of the United Nations. (PDF 583 kb)
Additional file 2: Literature review methodology and results. (DOCX 40 kb)

## Abbreviations

LMIC: Low- and middle-income countries
TDR: Special programme for research and training in tropical diseases
UN: United Nations
UNDP: United Nations Development Programme
UNICEF: United Nations Children’s Fund
VBD: Vector borne diseases
WHO: World Health Organization
