# Supplementary material for: Priorities and needs for research on urban interventions targeting vector-borne diseases: rapid review of scoping and systematic reviews
Source: Infect Dis Poverty. 2016 Dec 1;5:104. doi: 10.1186/s40249-016-0198-6 (PMC5131554; doi:10.1186/s40249-016-0198-6)
Supplement: Additional file 1: — Multilingual abstracts in the five official working languages of the United Nations. (PDF 583 kb) [file 40249_2016_198_MOESM1_ESM.pdf]

Translation of the abstract into the five official working languages of the United Nations

الأولويات والاحتياجات للبحث عن التدخلات الحضرية التي تستهدف الأمراض التي تنقلها الحشرات: استعراض سريع للفحص والمراجعات المنهجية

كلارا بيرموديز- تامايو، أوليف موكامانا، مابل كارابالي، ليدا أوزوريو، فلورنس فورنيه، كانبوبرك دابيريه، سيلينا تورتشي مارتيللي، أدولفو كونتريراس، فاليري ريدي

الملخص

يُبْرِز هذا البحث الأهمية الحاسمة للدليل على الوقاية من الأمراض التي تنقلها الحشرات (VBD) وتدخلات المكافحة في المناطق الحضرية عند تقييم الاحتياجات الحالية والمستقبلية، وذلك بهدف تحديد أولويات السياسة التي تعزز الخدمات الصحية الحضرية الشاملة والعادلة. يجب أن يقدم البحث المعرفة حول السياسات والتدخلات التي تهدف إلى مراقبة ومنع الأمراض التي تنقلها الحشرات (VBDs) على مستوى السكان والحد من عدم المساواة. وتشمل هذه التدخلات نهج السياسات والبرامج وتوزيع الموارد لمعالجة المحددات الاجتماعية للصحة وبسط نفوذها على المستويات التنظيمية والنظام.

Translated from English version into Arabic by free bird, through

针对城市媒传疾病干预的研究需求和优先领域：对勘域综述和系统综述的快速回顾

Clara Bermudez-Tamayo, Olive Mukamana, Mabel Carabali, Lyda Osorio, Florence Fournet, Kounbobr Roch Dabiré, Celina Turchi Marteli, Adolfo Contreras, Valéry Ridde

摘要

为促进城市卫生服务的包容性和平等性，需要评估现有的和将来的需求以制定政策优先领域。本文强调了此种情况下针对媒传疾病防控方面的循证是非常重要的。在群体水平防控媒传疾病并减少不公需要开展研究以产生政策和干预方面的知识。这些干预包括探索卫生的社会决定因素的政策、项目和资源分配策略，并且要在机构和系统水平发挥影响。

Translated from English version into Chinese by Men-Bao Qian, edited by Pin Yang, through

Priorités et besoins de recherche sur des interventions en milieu urbain ciblant des maladies vectorielles : bref examen des revues exploratoires et systématiques

Clara Bermudez-Tamayo, Olive Mukamana, Mabel Carabali, Lyda Osorio, Florence Fournet, Kounbobr Roch Dabiré, Celina Turchi Marteli, Adolfo Contreras, Valéry Ridde

RÉSUMÉ

Cet article souligne l'importance de données factuelles sur les interventions de prévention et de lutte contre les maladies vectorielles dans le milieu urbain lors de l'évaluation de besoins actuels et futurs avec pour objectif d'établir des priorités stratégiques qui promeuvent les services de santé urbains inclusifs et équitables. Les recherches doivent produire des connaissances à propos des politiques et des interventions conçues pour contrôler et prévenir des maladies vectorielles au niveau de la population et de réduire les inégalités. De telles interventions comprennent des politiques, des programmes et des approches de répartition des ressources qui abordent les déterminants sociaux de la santé et exercent une influence aux niveaux de l'organisation et du système.

Translated from English version into French by eric ragu, through

TRANSLATORS
WITHOUT BORDERS

Приоритеты и потребности городских мероприятий, направленных на борьбу с трансмиссивными болезнями: быстрый обзор подлежащих изучению вопросов и систематический обзор

Клара Бермудез-Тамайо, Олив Мукамана, Мэйбл Карабали, Лида Осорио, Флоренс Фурне, Коунбобр Роч Дабирэ, Селина Турки Мартели, Адольфо Контрерас, Валери Ридд

КРАТКИЙ ОБЗОР

Данный доклад подчеркивает важность мероприятий по предупреждению и контролю трансмиссивных болезней (ТБ) в городских условиях при оценке текущих и будущих потребностей, с целью установить в качестве приоритета всеобщий охват и равноправие при получении услуг здравоохранения. Исследование направлено на распространение знаний о действиях и мероприятиях, направленных на контроль и предупреждение трансмиссивных болезней у населения и на снижение неравенства в оказании услуг здравоохранения. Такие мероприятия включают распространение методов, программ и ресурсов, исследующих социальные детерминанты здоровья и оказывающих влияние на здравоохранения на организационном и системном уровнях.

Translated from English version into Russian by Natallia Lupik, through

Prioridades y necesidades de investigación en las intervenciones urbanas de lucha contra enfermedades transmitidas por vectores: examen rápido de la determinación del alcance y de las revisiones sistemáticas

Clara Bermudez-Tamayo, Olive Mukamana, Mabel Carabali, Lyda Osorio, Florence Fournet, Kounbobr Roch Dabiré, Celina Turchi Marteli, Adolfo Contreras, Valéry Ridde

RESUMEN

Este documento pone de relieve la importancia crítica de la evidencia sobre la prevención y las intervenciones de control de las enfermedades transmitidas por vectores (VBD) en los entornos urbanos a la hora de evaluar las necesidades actuales y futuras, con miras a establecer prioridades de las políticas que promueven los servicios de salud urbanas inclusivos y equitativos. La investigación debe producir conocimiento acerca de las políticas e intervenciones que tienen por objeto controlar y prevenir las VBD a nivel de la población y reducir las desigualdades. Estas intervenciones incluyen enfoques de políticas, programas y distribución de recursos que aborden los determinantes sociales de la salud y ejercen influencia en los niveles de organización y del sistema.

Translated from English version into Spanish by Susana Rosselli, through
