# Supplementary material for: Priorities and needs for research on urban interventions targeting vector-borne diseases: rapid review of scoping and systematic reviews
Source: Infect Dis Poverty. 2016 Dec 1;5:104. doi: 10.1186/s40249-016-0198-6 (PMC5131554; doi:10.1186/s40249-016-0198-6)
Supplement: Additional file 2: — Literature review methodology and results. (DOCX 40 kb) [file 40249_2016_198_MOESM2_ESM.docx]

**Appendix 1. Literature review methodology and results**

We searched Ovid MEDLINE, PubMed and the Cochrane library using various combinations of medical subject heading (MeSH) terms and keywords as well as personalized search strategies for each theme. The review was limited to studies published in English and French, and no time limits were applied on the searches.

The identification and further selection of papers were performed by one reviewer and one verifier (1). Systematic and scoping reviews were the focus of the searches, as their methodology allowed us to explore the extent or nature of evidence and to analytically interpret the reviewed literature and assess its quality (2).

**Theme 1: Vector-borne disease transmission dynamics in urban settings: A review of entomological and epidemiological issues and research**

For this theme, we used several combinations of MeSH terms and keywords according to the requirements of each of the searched databases. These include: “vector-borne disease”, “communicable diseases”, “transmission”, “disease transmission”, “transmission dynamics”, “urban population”, “urban setting*”, “urban area*”, “epidemiology*”, “epidemiological issues”, “entomological issues”, and “entomology*”. We identified one systematic review that studied the effect of urbanization on malaria transmission and the burden of this disease in Africa ([3](#_ENREF_1)). We also found that the Rapid Urban Malaria Project (RUMA) project, undertaken to systematically study key malariological features (4-8), made it possible to describe transmission patterns in sub-Saharan African countries: Abidjan (Côte d'Ivoire), Ouagadougou (Burkina Faso), Cotonou (Benin), and Dar es Salaam (United Republic of Tanzania). This search also yielded several literature reviews ([9-12](#_ENREF_2)), as well as other systematic reviews ([3-14](#_ENREF_7)) and a scoping review ([16](#_ENREF_10)) that studied subjects similar to this theme but did not specifically target urban settings.

**Theme 2: Vector-borne and other infectious diseases of poverty in urban settings: A comparative perspective on the evolution of urbanization trends (with a focus on the past 10-20 years and projections into the future)**

Searching the same databases and using several combinations of the following search terms: “vector-borne disease”, “infectious disease”, “communicable disease”, “urban*”, “urbanization, evolution of urbanization”, we found three relevant reviews: a scoping review that summarized the impact of urbanization on the epidemiology of infectious diseases in tropical countries and the emergence of infectious diseases at several times in these countries ([1](#_ENREF_11)7); a review mapping the global distribution of four dengue virus types throughout the 1943–2013 period and describing the impact of urbanization on this distribution in some areas ([18](#_ENREF_12)); and a literature review of the impact of urbanization on the biology of hosts, pathogens, and vectors ([19](#_ENREF_13)).

**Theme 3: Social dynamics and governance issues influencing urban VBD control, with an emphasis on intersectoral planning, communication and action, and community-oriented services**

Using combinations of the search terms “urban*”, “urban population”, “urban health”, “urban health services”, “vector-borne disease”, “communicable disease control”, “social environment” or “social dynamics”, “public health”, “health policy”, “inter-sectoral planning”, “health planning”, “community-based” and “community-oriented”, we found two reviews: a systematic review of studies done in both urban and rural areas and aimed at improving child immunization coverage in low- and middle-income countries ([20](#_ENREF_14)); and a literature review assessing the evidence for the effect of urban living on population health, related challenges, and directions for research and practice in urban health ([21](#_ENREF_15)) .

**Theme 4: Housing, water and sanitation: Issues in and solutions for infectious disease prevention and control**

We used several combinations of the following search terms: “infectious disease”, “communicable diseases”, “urban health”, “urban population”, “urban health services”, “urban*”, “housing”, “water”, and “sanitation”. Two systematic reviews of studies of interventions to prevent diarrhea were found. One consisted of interventions to improve the disposal of human feces in both rural and urban areas ([22](#_ENREF_16)), and the other concerned handwashing promotion interventions that took place mainly in rural areas ([23](#_ENREF_17)). A literature review of the impact of human activities on mosquito-borne diseases in urban areas of the Southeast Asia region was also identified ([24](#_ENREF_18)).

**Theme 5: Implementation research findings on infectious disease prevention and control in urban settings: Issues and solutions in large-scale interventions**

The following search terms were used: “evidence-based practice”, “program development”, “research”, “implementation research”, “prevention”, “primary prevention”, “secondary prevention”, “tertiary prevention”, “intervention”, “large-scale intervention”, “disease control”, “infectious diseases”, “communicable diseases”, “urbanization”, “urban setting”, “urban population”, and “urban*”. This search yielded two scoping reviews. One summarized historical approaches in research and interventions targeting infectious diseases, mainly water-related diseases, but not specific to urban areas ([2](#_ENREF_19)5), and the other used the social determinants of health model to examine key issues related to the prevention and control of HIV/AIDS, tuberculosis, and vector-borne diseases such as malaria and dengue fever in urban informal settlements ([27](#_ENREF_20)).

**Theme 6: VBD surveillance and community-based risk communication in urban areas. Interventions on health promotion and VBD**

Using the search terms: “communicable diseases”, “vector-borne disease”, “insect vectors”, “disease vectors”, “malaria”, “dengue”, “Culicidae”, “Aedes”, “Ixodes”, “disease outbreaks”, “surveillance”, “public health surveillance”, “communicable disease control”, “communication”, “risk communication”, “urban population”, and “urban areas”, two relevant literature reviews were found. One discussed the emergence of visceral leishmaniasis in urban areas of Brazil and challenges related to its control ([4](#_ENREF_3)), and the other reviewed urban zoonoses caused by Bortonella, Coxiella, Ehrlichia, and Rickettsia species, focusing on their occurrence in the United States of America (USA) ([27](#_ENREF_21)).

Other search terms used to explore reviews that studied this theme included: “health promotion or healthy people programs”, “preventive health services”, “disease outbreaks”, “communicable diseases”, “disease vectors”, “insect vectors”, “Aedes” , “Culidae”, “Dengue”, “malaria”, “vector-borne diseases”, “urban population”, and “urban setting”. Two reviews were obtained: a systematic review of handwashing promotion interventions to prevent diarrhea that took place mainly in rural areas ([22](#_ENREF_17)) (also identified in theme 4), and a literature review that examined the impact of correctional systems (jails and prisons) on the health of urban populations in the USA ([28](#_ENREF_22)).

The following search terms were also used to search for reviews relevant to the theme: “surveillance”, “disease control”, “control program”, “monitoring”, “vector*”, “vector-borne disease”, “dengue”, “malaria”, “leishmaniasis”, “filariasis”, “urban*”, “urban vector”, “urban setting”, “urban population”, and “urban area”. This search yielded a systematic review of studies of various mosquito larval source management strategies for the control of malaria conducted in urban and rural areas ([14](#_ENREF_9)). Four literature reviews were also found: the first highlighted the difficulties related to existing entomological surveillance tools for dengue vectors and the need for improved and more reliable tools ([32](#_ENREF_26)); the second (also found in theme 5) discussed the urbanization of visceral leishmaniasis in Brazil and strategies to control the disease ([5](#_ENREF_3)); the third reviewed various malaria control strategies and innovations in disease vector control, as well as challenges related to malaria eradication ([33](#_ENREF_27)); and the fourth discussed the challenges of dengue prevention in Southeast Asia and a regional surveillance approach for active dengue virus in urban areas ([34](#_ENREF_28)). The search yielded other literature reviews conducted in the 1990s, which discussed dengue vector control approaches ([35](#_ENREF_29), [36](#_ENREF_30)) and control methods for leishmaniasis ([37](#_ENREF_31)-39,) in various areas (urban and rural).

**Theme 7: Population dynamics and their interaction with social determinants of health**

Focusing this theme on three vector-borne diseases—malaria, dengue, and leishmaniasis—we used several combinations of the following search terms: “population dynamics”, “migration”, “population movement”, “human migration”, “population growth”, “social determinants of health”, “social determinant*”, “determinants of health”, “malaria”, “dengue”, “dengue virus”, “leishmaniasis”, “vector-borne disease”, and “vector-borne*”; no relevant reviews were identified.

The studies identified through this review focused mainly on malaria and dengue. Few studies looked at other VBDs such as leishmaniasis. The majority of the scoping and systematic reviews identified studied vector-borne or infectious diseases in both urban and rural areas and did not focus particularly on urban settings. Furthermore, as mentioned, many literature reviews studied subjects similar to the themes suggested in this call but were characterized by a lack of clarity regarding the methods used to conduct them. Their methodological quality can therefore be considered below that of scoping or systematic reviews, which would use clear and comprehensive methodology to study the suggested themes.

**References**

1. Tricco AC, Antony J, Zarin W, Strifler L, Ghassemi M, Ivory J, et al. A scoping review of rapid review methods. BMC Med. 2015;13:224.
2. Ganann R, Ciliska D, Thomas H. Expediting systematic reviews: methods and implications of rapid reviews. Implement Sci. 2010;5:56.
3. Hay SI, Guerra CA, Tatem AJ, Atkinson PM, Snow RW. Urbanization, malaria transmission and disease burden in Africa. Nat Rev Microbiol. 2005;3:81-90.
4. Wang SJ, Lengeler C, Smith TA, Vounatsou P, Akogbeto M, Tanner M. Rapid Urban Malaria Appraisal (RUMA) IV: epidemiology of urban malaria in Cotonou (Benin). Malar J. 2006;2;5:45.
5. Wang SJ, Lengeler C, Mtasiwa D, Mshana T, Manane L, Maro G, et al. Rapid Urban Malaria Appraisal (RUMA) II: epidemiology of urban malaria in Dar es Salaam (Tanzania). Malar J. 2006;5:28.
6. Wang SJ, Lengeler C, Smith TA, Vounatsou P, Cissé G, Tanner M. Rapid Urban Malaria Appraisal (RUMA) III: epidemiology of urban malaria in the municipality of Yopougon (Abidjan). Malar J. 2006;5:29.
7. Wang SJ, Lengeler C, Smith TA, Vounatsou P, Diadie DA, Pritroipa X, et al. Rapid urban malaria appraisal (RUMA) I: epidemiology of urban malaria in Ouagadougou. Malar J. 2005;4:43.
8. Wang SJ, Lengeler C, Smith TA, Vounatsou P, Cissé G, Diallo DA, et al. Rapid urban malaria appraisal (RUMA) in sub-Saharan Africa. Malar J. 2005;4:40.
9. Guzmán-Bracho C. Epidemiology of Chagas disease in Mexico: an update. Trends Parasitol. 2001;17:372-6.
10. Harhay MO, Olliaro PL, Costa DL, Costa CHN. Urban parasitology: visceral leishmaniasis in Brazil. Trends Parasitol. 2011;27:403-9.
11. Simonsen PE, Mwakitalu ME. Urban lymphatic filariasis. Parasitol Res. 2013;112:35-44.
12. Jansen CC, Beebe NW. The dengue vector Aedes aegypti: what comes next. Microbes Infect. 2010;12:272-9.
13. Mondet B, da Rosa A, Vasconcelos P. [The risk of urban yellow fever outbreaks in Brazil by dengue vectors. Aedes aegypti and Aedes albopictus]. Bull Soc Pathol Exot. 1996;89:107-13; discussion 14.
14. González U, Pinart M, Sinclair D, Firooz A, Enk C, Vélez ID, et al. Vector and reservoir control for preventing leishmaniasis. Cochrane Database Syst Rev. 2015;8:CD008736.
15. Pluess B, Tanser FC, Lengeler C, Sharp BL. Indoor residual spraying for preventing malaria. Cochrane Database Syst Rev. 2010;4:CD006657.
16. Tusting LS, Thwing J, Sinclair D, Fillinger U, Gimnig J, Bonner KE, et al. Mosquito larval source management for controlling malaria. Cochrane Database Syst Rev. 2013;8:CD008923.
17. Chanda E, Govere JM, Macdonald MB, Lako RL, Haque U, Baba SP, et al. Integrated vector management: a critical strategy for combating vector-borne diseases in South Sudan. Malar J. 2013;12:369.
18. Alirol E, Getaz L, Stoll B, Chappuis F, Loutan L. Urbanisation and infectious diseases in a globalised world. Lancet Infect Dis. 2011;11(2):131-41.
19. Messina JP, Brady OJ, Scott TW, Zou C, Pigott DM, Duda KA, et al. Global spread of dengue virus types: mapping the 70 year history. Trends Microbiol. 2014;22:138-46.
20. Bradley CA, Altizer S. Urbanization and the ecology of wildlife diseases. Trends Ecol Evol. 2007;22:95-102.
21. Oyo-Ita A, Nwachukwu CE, Oringanje C, Meremikwu MM. Interventions for improving coverage of child immunization in low-and middle-income countries. Cochrane Database Syst Rev. 2011;7:CD008145.
22. Galea S, Vlahov D. Urban health: evidence, challenges, and directions. Annu Rev Public Health. 2005;26:341-65.
23. Clasen TF, Bostoen K, Schmidt WP, Boisson S, Fung IC, Jenkins MW, et al. Interventions to improve disposal of human excreta for preventing diarrhoea. Cochrane Database Syst Rev. 2010;6:CD007180.
24. Ejemot-Nwadiaro RI, Ehiri JE, Arikpo D, Meremikwu MM, Critchley JA. Hand washing promotion for preventing diarrhoea. Cochrane Database Syst Rev. 2015;9:CD004265.
25. Bang Y, Shah N. Human ecology related to urban mosquito-borne diseases in countries of South East Asia region. J Commun Dis. 1988;20:1-17.
26. Batterman S, Eisenberg J, Hardin R, Kruk ME, Lemos MC, Michalak AM, et al. Sustainable control of water-related infectious diseases: a review and proposal for interdisciplinary health-based systems research. Environ Health Perspect. 2009;117:1023-32.
27. David A, Mercado S, Becker D, Edmundo K, Mugisha F. The prevention and control of HIV/AIDS, TB and vector-borne diseases in informal settlements: challenges, opportunities and insights. J Urban Health. 2007;84:65-74.
28. Comer JA, Paddock CD, Childs JE. Urban zoonoses caused by Bartonella, Coxiella, Ehrlichia, and Rickettsia species. Vector Borne Zoonotic Dis. 2001; 1:91-118.
29. Freudenberg N. Jails, prisons, and the health of urban populations: a review of the impact of the correctional system on community health. J Urban Health. 2001;78:214-35.
30. Dye C. After 2015: infectious diseases in a new era of health and development. Philos Trans R Soc Lond, B, Biol Sci. 2014;369:20130426.
31. Agyei-Mensah S, de-Graft Aikins A. Epidemiological transition and the double burden of disease in Accra, Ghana. J Urban Health. 2010;87:879-97.
32. Oni T, Unwin N. Why the communicable/non-communicable disease dichotomy is problematic for public health control strategies: implications of multimorbidity for health systems in an era of health transition. Int Health. 2015;7:390-9.
33. Sivagnaname N, Gunasekaran K. Need for an efficient adult trap for the surveillance of dengue vectors. Ind J Med Res. 2012;136:739-49.
34. Enayati A, Hemingway J. Malaria management: past, present, and future. Annu Rev Entomol. 2010;55:569-91.
35. Ooi EE, Gubler DJ. Dengue in Southeast Asia: epidemiological characteristics and strategic challenges in disease prevention. Cad Saude Publica. 2009;25 Suppl 1:S115-24.
36. Yap HH, Chong NL, Foo AE, Lee CY. Dengue vector control: present status and future prospects. Kaohsiung J Med Sci. 1994;10 Suppl:S102-8.
37. Gubler DJ, Clark GG. Community-based integrated control of Aedes aegypti: a brief overview of current programs. Am J Trop Med Hyg. 1993;50 Suppl 6:50-60.
38. Yaman M. [Control of phlebotomine sandflies and the latest development in this field]. Turkiye Parazitol Derg. 2007;32:280-7.
39. Maroli M, Khoury C. [Prevention and control of leishmaniasis vectors: current approaches]. Parassitologia. 2004;46:211-5.
